# Supplementary material for: Prebiotic Effect of Polysaccharides and Flavonoids from Passiflora foetida Fruits on the Human Intestinal Microbiota Associated with Obesity
Source: Foods. 2025 Sep 17;14(18):3222. doi: 10.3390/foods14183222 (PMC12469676; doi:10.3390/foods14183222)
Supplement: Supplementary file 1 [file foods-14-03222-s001.zip › foods-3819864-supplementary.pdf]

Table S1

Primers and amplification conditions for real-time PCR assays.

Bacterial group	Primers	Primer sequence and direction (5′–3′) [a]	Product size (bp)	Annealing T (°C)	Time (s)
Akkermansia	AM1-F	F: 5’ CAGCACGTGAAGGTGGGGAC 3’	329	58	40
	AM2-R	R: 5’ CCTTGCGGTTGGCTTCAGAT 3’			
Alistipes	AlstpF	F: 5’ GTACTAATTCCCCATAACATTCGAG 3’	83	60	40
	AlstpR	R: 5’ CTAATACAACGCATGCCCATCTT 3’			
Archaea	A-751F	F: 5’ CCGACGGTGAGRGRYGAA 3’	287	55	40
	A-976R	R: 5’ YCCGGCGTTGAMTCCAATT 3’			
Atopobium	AtopF	F: 5’ GGGTTGAGAGACCGACC 3’	196	55	40
	AtopR	R: 5’ CGGRGCTTCTTCTGCAGG 3’			
B coccoides	Erec-F	F: 5’ CGGTACCTGACTAAGAAGC 3’	429	55	40
	Erec-R	R: 5’ AGTTTYATTCTTGCGAACG 3’			
Bacteroides	Bac303F	F: 5’ GAAGGTCCCCCACATTG 3’	103	60	40
	Bfr-Fmrev	R: 5’ CGCKACTTGGCTGGTTCAG 3’			
Bifidobacterium	Bif-F	F: 5’ CTCCTGGAAACGGGTGG 3’	593	55	40
	Bif-R	R: 5’ GGTGTTCTTCCCGATATCTACA 3’			
Bilophia	BwF	F: 5’ AAGTCCTTCGGGGCGAGTAA 3’	239	60	40
	BwR	R: 5’ ATCCTCTCAGACCGGCTAC 3’			
C leptum	sg-Clept-F	F: 5’ GCACAAGCAGTGGAGT 3’	239	55	40
	sg-Clept-R	R: 5’ CTTCCTCCGTTTTGTCAA3’			
Enterobacteriaceae	F-ent	F: 5’ ATGGCTGTCGTCAGCTCGT 3’	385	55	40
	R-ent	R: 5’ CCTACTTCTTTTGCAACCCACTC3’			
Enterococcus	g-Encoc-F	F: 5’ ATCAGAGGGGGATAACACTT 3’	336	55	40
	g-Encoc-R	R: 5’ ACTCTCATCCTTGTTCTTCTC 3’			
Faecalibacterium	Fprau 07	F: 5’ CCATGAATTGCCTTCAAAACTGTT 3’	192	60	40
	Fprau 02	R: 5’ GAGCCTCAGCGTCAGTTGGT 3’			

Lactobacillus	Lab-F362<br>Lab-R677	F: 5’ AGCAGTAGGGAATCTTCCA 3’<br>R: 5’ CACCGCTACACATGGAG 3’	341	58	40
Prevotella	g-Prevo-F<br>g-Prevo-R	F: 5’ CACRGTAAACGATGGATGCC 3’<br>R: 5’ GGTCGGGTTGCAGACC 3’	528	60	40
Roseburia	RosF1<br>RosR1	F: 5’ GCGGTRCGGCAAGTCTGA 3’<br>R: 5’ CCTCCGACACTCTAGTMCGAC 3’	81	60	40
Ruminococcus	Rflbr730F<br>Clep866mR	F: 5’ GGCGGCYTRCTGGGCTTT 3’<br>R: 5’ CCAGGTGGATWACTTATTGTGTTAA 3’	157	60	40
SRB	DSR1F+<br>DSR-R	F: 5’ ACSCACTGGAAGCACGGCGG 3’<br>R: 5’ GTGGMRCCGTGCAKRTTGG 3’	221	60	40

[a] F, forward; R, reverse.

Table S2

Chemical composition of PFP.

Carbohydrate (%)	Protein (%)	Ash (%)	TFC (%)	TPC (%)	Uronic acid (%)	Molecular weight (Da)
65.23±3.05	0.56±0.02	1.45±0.09	12.64±1.15	4.27±0.28	17.05±2.71	5.24±0.28

Table S3

Monosaccharide composition of PFP.

Monosaccharide	Fuc	Ara	Gal	Glc	Xyl	Man	GalA	GlcA

Molar ratio, %	1.37±0.09	14.87±0.15	26.63±0.31	9.26±0.24	7.44±0.28	9.30±0.13	28.44±0.14	2.68±0.20

Table S4
Analysis of the chemical constituents of PFF by UHPLC-LTQ–Orbitrap.

Classification	No.	RT (min)	Compound name	Molecular formula	Ion peak	Fragment ions	Relative abundance (%)
Alkaloids	1	1.68	Stachydrine	$C_7H_{13}NO_2$	144.1020 $[M+H]^+$	116.9719, 121.9664, 84.0808	0.65
	2	2.23	Uridine	$C_9H_{12}N_2O_6$	243.0618 $[M-H]^-$	200.0556, 152.0341, 140.0341, 110.0233	0.38
	3	2.25	Nicotinamide	$C_6H_6N_2O$	123.0553 $[M+H]^+$	72.9372, 80.0495, 96.0444	0.74
	4	2.33<br>2.34	Isoguanosine	$C_{10}H_{13}N_5O_5$	282.0842 $[M-H]^-$<br>284.0988 $[M+H]^+$	150.0409<br>152.0568	0.82
	5	5.03	Gramine	$C_{11}H_{14}N_2$	175.1230 $[M+H]^+$	133.1012, 119.0856, 82.0216	0.69
	6	5.72	L-Tryptophan	$C_{11}H_{12}N_2O_2$	205.0971 $[M+H]^+$	159.0919, 149.0601, 146.0601, 118.0651	3.53

	7	6.93	Aconine	$C_{25}H_{41}NO_9$	500.2853 [M+H]$^+$	354.1655, 258.1084, 147.0765	0.19
	8	19.86	Mitraphylline	$C_{21}H_{24}N_2O_4$	369.1802 [M+H]$^+$	238.0830, 130.0860	0.06
	9	9.20<br>9.22	Fisetin	$C_{15}H_{10}O_6$	285.0403 [M-H]$^-$<br>287.0547 [M+H]$^+$	270.0536, 149.0228, 135.0074<br>241.0498, 224.7761, 147.0439	2.45
	10	11.49	Tectorigenin	$C_{16}H_{12}O_6$	299.0559 [M-H]$^-$	284.0327	10.44
	11	11.54	Iristectorigenin B	$C_{17}H_{14}O_7$	331.0809 [M+H]$^+$	316.0579	7.02
Flavones	12	13.56<br>13.57	Genkwanin	$C_{16}H_{12}O_5$	283.0612 [M-H]$^-$<br>285.0754 [M+H]$^+$	268.0379<br>270.0520, 242.0575	5.85
	13	13.58<br>13.59	Glycitein	$C_{18}H_{14}O_8$	283.0612 [M-H]$^-$<br>285.0754 [M+H]$^+$	268.0379<br>270.0520, 242.0575，	5.85
	14	14.49	Pectolinarigenin	$C_{17}H_{14}O_6$	313.0718 [M-H]$^-$	298.0482, 283.0246, 255.02791	3.51
	15	14.60<br>14.61	Lysionotin	$C_{18}H_{16}O_7$	343.0821 [M-H]$^-$<br>345.0964 [M+H]$^+$	328.0590, 313.0355<br>330.0733, 315.05054	1.11
	16	17.02	Tricin	$C_{17}H_{14}O_7$	329.2332 [M-H]$^-$	314.0434, 229.1441, 211.1332, 171.1016	0.05
Flavonols	17	4.19	Silibinin	$C_{25}H_{22}O_{10}$	481.1119 [M-H]$^-$	474.2255, 125.0113	0.06
	18	7.91	Dihydromyricetin	$C_{15}H_{12}O_8$	321.0602 [M+H]$^+$	153.0547, 139.0390	0.19
	19	10.18<br>10.20	Taxifolin	$C_{15}H_{12}O_7$	303.0510 [M-H]$^-$<br>305.0656 [M+H]$^+$	285.0405, 275.0550, 241.0500, 177.0185<br>287.0540, 259.0600, 231.0649, 153.0183	0.20

Flavonoid glycoside	20	6.18	Vaccarin	$C_{32}H_{38}O_{19}$	725.1942 [M-H]$^-$	635.1609, 605.1560, 545.1312, 473.1072	0.30
	21	6.58	Tinnevellin glucoside	$C_{20}H_{24}O_{9}$	407.1347 [M-H]$^-$	363.0176, 232.9749, 218.9799, 143.0338	0.06
	22	7.74	Schaftoside	$C_{26}H_{28}O_{14}$	563.1406 [M-H]$^-$	545.1305, 473.1094, 443.0986, 353.0667	7.25
		7.76			565.1551 [M+H]$^+$	547.1445, 475.1106, 445.1132, 379.0811	
	23	7.76	2''-O-β-L-galactopyranosylorientin	$C_{27}H_{30}O_{16}$	609.1464 [M-H]$^-$	489.1041, 429.0827, 357.0616, 327.0510	3.15
		7.78			611.1604 [M+H]$^+$	449.1079, 431.0974, 413.0872, 329.0656	
	24	8.07	Leucoside	$C_{26}H_{28}O_{15}$	579.1361 [M-H]$^-$	429.0825, 357.0615, 327.0511, 309.0406	0.59
		8.09			581.1502 [M+H]$^+$	431.1003, 359. 0834, 329.0653, 287.0557	
	25	8.26	Orientin	$C_{21}H_{20}O_{11}$	447.0933 [M-H]$^-$	327.0511, 285.0468, 268.0412, 257.0502	2.87
		8.28			449.1078 [M+H]$^+$	329.0655, 287.0561, 270.0485, 259.0612	
	26	8.37	Aurantio-obtusin β-D-glucoside	$C_{23}H_{24}O_{12}$	491.1194 [M-H]$^-$	476.0975, 445.1731, 329.0668, 314.0433	0.22
		8.39			493.1338 [M+H]$^+$	402.2362, 331.0811, 316.0564	
	27	8.40		$C_{27}H_{30}O_{15}$	593.1511 [M-H]$^-$	473.1090, 413.0880, 311.0561, 293.0456	10.16

		8.42	4'-O-Glucosylvitexin		595.1655 [M+H]$^+$	475.1223, 433.1130, 415.1020, 313.0705	
	28	8.42	Vicenin II	$C_{27}H_{30}O_{15}$	593.1511 [M-H]$^-$	473.1081, 413.0880, 311.0561, 293.0456	15.26
		8.44			595.1655 [M+H]$^+$	475.1223, 433.1130, 415.1020, 313.0705	
	29	8.44	Diosmetin-7-O-β-D-glucopyranoside	$C_{22}H_{22}O_{11}$	461.1092 [M-H]$^-$	431.2272, 299.0561, 284.0325	1.29
		8.46			463.1232 [M+H]$^+$	445.2439, 301.0707, 286.0465	
	30	8.78	Rhoifolin	$C_{27}H_{30}O_{14}$	577.1566 [M-H]$^-$	413.0879, 311.0558, 293.0457	0.14
		8.80			579.1705 [M+H]$^+$	433.1131, 415.1022, 397.0918, 313.0706	
	31	9.15	Apigenin-7-O-β-D-glucoside	$C_{21}H_{20}O_{10}$	431.0982 [M-H]$^-$	413.1839, 341.0666, 311.0562, 269.0458	3.57
		9.17			433.1127 [M+H]$^+$	415.1027, 397.0917, 313.0705, 271.0593	
	32	9.53	Myricitrin	$C_{21}H_{20}O_{12}$	463.0885 [M-H]$^-$	417.2466, 343.2127, 301.0718, 285.0398	0.06
Phenols	33	7.22	Vanillic acid	$C_8H_8O_4$	169.0496 [M+H]$^+$	151.0390, 125.0598, 111.0441, 93.0336,	0.32
	34	7.52	Octyl gallate	$C_{15}H_{22}O_5$	281.1393 [M-H]$^-$	237.1492, 171.1170, 123.0802	4.39
	35	9.86	Salicylic acid	$C_7H_6O_3$	137.0231 [M-H]$^-$	135.0075, 109.0281, 93.0331	0.65

Terpenoids	36	6.96	Camphor	$C_{10}H_{16}O$	153.1273 $[M+H]^+$	135.1169, 107.0856, 93.0699, 81.0699	1.66
	37	7.54	Abscisic acid	$C_{15}H_{20}O_4$	265.1433 $[M+H]^+$	247.1326, 203.1066, 175.0755, 135.0804	3.40
Fatty acid	38	20.52	Linolenic acid	$C_{18}H_{30}O_2$	279.2315 $[M+H]^+$	167.0343, 149.0234, 95.0855, 67.0544	0.32
Coumarin	39	12.71	7-Methoxycoumarin	$C_{10}H_8O_3$	177.0545 $[M+H]^+$	149.0599, 145.0285, 130.0651, 117.0335	0.56
